# Diet-Derived Antioxidants and Their Role in Inflammation, Obesity and Gut Microbiota Modulation

**DOI:** 10.3390/antiox10050708

**Published:** 2021-04-29

**Authors:** Andrea Deledda, Giuseppe Annunziata, Gian Carlo Tenore, Vanessa Palmas, Aldo Manzin, Fernanda Velluzzi

**Affiliations:** 1 Obesity Unit, Department of Medical Sciences and Public Health, University of Cagliari, Ospedale San Giovanni di Dio, Via Ospedale 54, 09124 Cagliari, Italy; andredele@tiscali.it (A.D.); fernandavelluzzi@gmail.com (F.V.); 2 Department of Pharmacy, University of Naples Federico II, Via Domenico Montesano 49, 80131 Naples, Italy; giancarlo.tenore@unina.it; 3 Microbiology and Virology Unit, Department of Biomedical Sciences, University of Cagliari, Cittadella Universitaria di Monserrato, 09042 Monserrato, Italy; vanessapalmas@hotmail.it (V.P.); aldomanzin@unica.it (A.M.)

**Keywords:** inflammation, obesity, gut microbiota, oxidative stress, antioxidants, polyphenols, ageing, nutraceutical

## Abstract

It is generally accepted that gut microbiota, inflammation and obesity are linked to the development of cardiovascular diseases and other chronic/non-communicable pathological conditions, including cancer, neurodegenerative diseases and ageing-related disorders. In this scenario, oxidative stress plays a pivotal role. Evidence suggests that the global dietary patterns may represent a tool in counteracting oxidative stress, thus preventing the onset of diseases related to oxidative stress. More specifically, dietary patterns based on the regular consumption of fruits and vegetables (i.e., Mediterranean diet) have been licensed by various national nutritional guidelines in many countries for their health-promoting effects. Such patterns, indeed, result in being rich in specific components, such as fiber, minerals, vitamins and antioxidants, whose beneficial effects on human health have been widely reported. This suggests a potential nutraceutical power of specific dietary components. In this manuscript, we summarize the most relevant evidence reporting the impact of dietary antioxidants on gut microbiota composition, inflammation and obesity, and we underline that antioxidants are implicated in a complex interplay between gut microbiota, inflammation and obesity, thus suggesting their possible role in the development and modulation of chronic diseases related to oxidative stress and in the maintenance of wellness. Do all roads lead to Rome?

## 1. Introduction

Vegetables, particularly if they do not receive chemical/technological treatments, contain large amounts of substances with antioxidant properties that are capable of reducing the negative effects of the redox processes which each cell undergoes due to its biochemical reactions. Therefore, vegetable-based diets, such as the Mediterranean diet [1], DASH [2] or even more restrictive ones such as vegetarian and vegan diets [3], are able to bring greater quantities of these substances than more common ones, such as the so called “cafeteria diet” or a Western diet often undertaken by most people. The aforementioned diets could be characterized by the presence of vegetable-derived foods which have lost some of their antioxidant properties and are rich in “empty” calories due to their refined/industrially modified form; moreover, the excess of animal-derived foods is linked to microbial alterations and the production of inflammatory metabolites (TMAO), contributing to the increased risk of obesity and so-called non-communicable diseases (cardiovascular diseases, diabetes, osteoarthritis, asthma, etc.) [4,5,6]. Processed food is also a source of additives that have a negative effect on the intestine, microbiota, liver and nervous system, mainly because of oxidative stress [7].

Fruits and vegetables are characterized by low calorie density and are high in micro nutrients [8].

**Figure 1:**
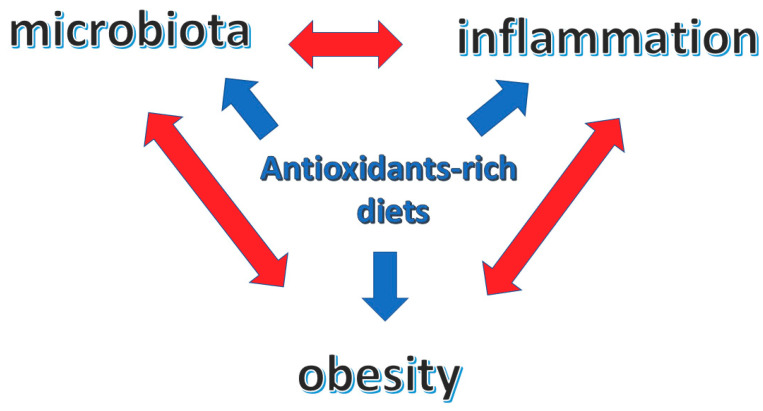
Central role of antioxidants-rich diets in the interplay between microbiota, inflammation and obesity.

Diet, dietary antioxidants, inflammation and body composition (obesity) are strictly interwoven with reciprocal–bidirectional relations that reflect on the overall health condition (Figure 1).

One of the great debates within nutrition concerns whether including animal foods is necessary or suitable for health purposes [9].

Recently the debate has been the subject of a publication which reported the different opinions on the matter. Many agreed on the fact that vegetable-based diets are associated with a lower body weight and reduced risk of diabetes; saturated fats and heme iron, typically present in animal-derived food, can be particularly harmful to part of the population; however, care must be taken of foods’ quality and their culinary use must be supervised in order not to lose nutrients and, most importantly, not to reduce the income of essential micro- and macronutrients, regardless of the diet type [9].

It is strongly advised that vegetables be unprocessed; therefore, whole grains, tubers, legumes, fruits and nuts are recommended. Moreover, the use of proteins of plant origin has recently been linked to a lower incidence of chronic diseases and lower mortality [10]. It is widely reported that polyphenols, a large family of phytochemicals mainly coming from plant-based foods, possess numerous beneficial biological activities and their prolonged intake exerts protective effects on health [11]. On this issue, a recent systematic review documented an inverse association between polyphenols or flavonoids intake and risk of diabetes, cardiovascular diseases and mortality, reporting also several promising effects on age-related diseases, but no effects on various types of cancer. Regarding subclasses of polyphenols, a wide range of flavonoids, including flavonols, anthocyanidins, proanthocyanidins, flavones, flavanones and flavan-3-ols, have been shown to be linked to reduced cardiovascular events and total mortality, but, due to the heterogeneity of studies, data are still conflicting and not definitive about the optimal dietary intake of these compounds. Nevertheless, a polyphenol-rich dietary pattern should be recommended [12].

Vegetables, but also fruits, whole grains, legumes and nuts, olive oil, and beverages (plant based) such as coffee and tea, are good plant-based sources of polyphenols because of their antioxidant properties [13,14]; to our knowledge, only honey can be considered a source of polyphenols with similar antioxidant activity [15].

It is well-known that most common diets, and especially the Western diet, have prioritized the use of animal-derived foods, which has led to a progressively reduced intake of vegetable-based foods. On the contrary, the Mediterranean diet is a good source of plant-based foods, and its key components, such as olive oil, nuts, red wine, legumes, fruits and vegetables, are all polyphenol-rich foods. Moreover, it has been suggested that the protective effects on health associated with a higher adherence to the Mediterranean diet, could be attributed to the anti-inflammatory, antioxidative and insulin-sensitizing properties of the bioactive components of this dietary pattern [16].

Moreover, current diets are rich in saturated fats and processed sources of carbohydrates, such as high fructose corn syrup, as well as salt, while they are deficient in fiber, vitamins and minerals. These diets are one of the main causes of increased obesity and associated chronic diseases, most of which are linked to a low-grade chronic inflammation. Today the question arises as to whether reducing or blocking the intake of such foods could lead to a "food as medicine" approach, i.e., favoring the body’s natural repairing and healing mechanisms through the removal of processed foods, which have been proven to be the main source for diseases, from diets [17].

Oxidative stress has been reported to be linked to several metabolic diseases, such as obesity, diabetes, hypertension or dyslipidemia, and the chronic exposure to elevated levels of glucose, triglycerides and free fatty acids seems to activate the oxidative process. In particular, hyperglycemia increases oxidative stress, encouraging glycation and oxidation of lipoproteins and production of adhesion molecules, and therefore creating the ideal environment for cardiovascular diseases. The consumption of foods with a high content of antioxidants and polyphenols increases plasma antioxidant capacity and decreases oxidative stress markers in subjects affected by metabolic syndrome and related conditions. Weight loss alone could increase antioxidant capacity; however, that might be encouraged by introducing a high dose of antioxidants, which causes a synergic effect on low-calorie diets [18].

Some chemical species, such as reactive oxygen and nitrogen species (ROS and RNS respectively), are free radicals and highly reactive unstable substances that are formed in vivo as a result of ordinary metabolic processes (mitochondrial oxidation, energy metabolism and virtually every biochemical reaction) or exposure to external factors like X-rays, UV rays, ozone, air pollutants, cigarette smoke, etc. Being unstable, free radicals tend to attack other molecular structures (including nucleic acids, proteins and lipids) to complete their octets and achieve chemical stability. When this process is not effectively counteracted, oxidative stress takes place. Antioxidants can inhibit oxidative stress mediated by free radicals and their toxic side effects in the human body. Some free radicals are, however, necessary for the proper functioning of the organism; therefore, a correct balance between reducing and oxidizing molecules (redox), as well as between reactive species and neutralizing elements, is the basis of health. Unbalanced redox systems and excessive oxidative stress are, along with chronic inflammation, among the main causes of non-communicable diseases, and between these two conditions, there is a close interdependence. Oxidative stress causes chronic inflammation, which, in turn, stimulates oxidative stress, in a vicious circle typical of chronic diseases [19].

The main endogenous antioxidant defenses are glutathione-peroxidase, catalase (enzymatic) and uric acid and bilirubin (non-enzymatic). However, the support given by exogenous molecules seems fundamental, especially as age progresses. Although some animal-derived antioxidants such as vitamin A and the anti-inflammatory carotenoid astaxanthin also exist [20], these molecules, including some vitamins, both hydro- (vitamin C) and fat-soluble (vitamin A in precursor forms and vitamin E) ones, carotenoids, polyphenols and various flavonoids, are all derived from vegetable sources [21]. It should, nevertheless, be taken into account that when used as exogenous supplements, antioxidants could reach supraphysiological doses that are capable of producing detrimental rather than beneficial effects, thus increasing the oxidative stress status. For this reason, different variables should be considered in antioxidants supplementation, such as the type or the combination of antioxidants, the dose and the duration of intake. On the contrary, a well-balanced diet naturally rich in antioxidants, such as the Mediterranean diet, particularly if combined with a regular physical activity, represents the best tool against oxidative stress [22,23].

Among the plant-derived bioactive compounds, polyphenols represent the largest class, and they include more than 8000 different molecules. Polyphenols are secondary metabolites produced by plants as protective agents against UV radiations, pathogen aggression and oxidative stress [24]. In humans, when prescribed as nutraceutical supplements and/or through dietary sources, they exert numerous properties, including antioxidant, anti-inflammatory [25], antidiabetic [24], anticancer [26,27], cardio-protective [28,29,30] and antiviral [31,32] activities.

### 1.1. Chemical Features and Bioavailability of Polyphenols

**Figure 2:**
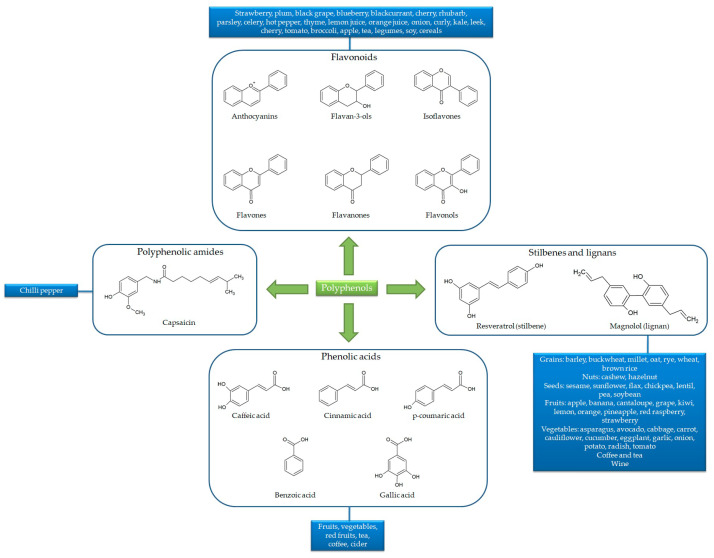
Chemical structures and sources of the main polyphenol classes.

Besides the numerous compounds, polyphenols share the same basic chemical structure built around one or more phenolic rings with hydroxyl groups. In general, polyphenol structures derive from phenylalanine (or from its precursor, shikimic acid), conjugated with one or more sugars, carboxylic, organic acid, amine, lipid or phenol residues via hydroxyl groups. Owing to their chemical structure, polyphenols are classified into flavonoids (including anthocyanins, flavones, flavanones, flavonols, isoflavones and flavan-3-ols), phenolic acids, polyphenolic amides and other polyphenol compounds (including stilbenes or lignans). Flavonoid basic structures consist of two aromatic rings (A and B) bound by three carbon atoms forming an additional oxygenated heterocycle (C ring). Conversely, phenolic acid, polyphenolic amide, stilbene and lignan structures consist of one main phenolic ring with one or more hydroxyl groups, carbon chains and aromatic rings [24] (Figure 2).

In plants, polyphenols are generally present in a glycosidic form that may affect their intestinal absorption, and thus the in vivo bioavailability. More specifically, polyphenol molecules are bound to fibers through glucose residues; during digestive processes, this glycosidic bond is generally broken, releasing the aglycone, which can then be absorbed. In addition, further central factors involved in gastrointestinal digestion, including physicochemical conditions (i.e., pH variation and digestive enzymes), intestinal permeation (i.e., difficult cell-membrane diffusion) and rapid excretion (i.e., rapid hepatic metabolism) also severely affect polyphenol bioavailability [24]. This results in the need to find new strategies (including innovative nutraceutical formulation and pharmaceutical technologies) aimed at dealing with this crucial feature of polyphenols and optimizing their health-promoting potential. Among these, microencapsulation has been licensed as one of the most effective strategy to enhance both bioaccessibility and bioavailability of polyphenols [24]. This is a technological process widely used in both the pharmaceutical and nutraceutical industries, with the scope to incorporate various compounds (i.e., liquid, solid or gaseous substances) into microscopic capsules consisting of different coating materials (i.e., polymers of lipids, proteins, or carbohydrates). This technique has been demonstrated to be a valid tool for delivering food-derived bioactive compounds, due to its ability to (i) protect them from degradation during the transit into the gastrointestinal tract, (ii) increase their water solubility and (iii) improve their permeation across the intestinal barrier. So far, various microencapsulation techniques have been developed, such as spray-drying, lyophilization, coacervation, ionic gelation and solvent evaporation. The choice of the more appropriate technique is based on the chemical features of the incorporating compounds [24].

### 1.2. Polyphenols and Health

Polyphenols have been shown to positively influence human health through different mechanisms. The anti-inflammatory activity of resveratrol, anthocyanins and curcumin has been associated to the protective effect against the chronic low-grade inflammation that is characteristic of Non-Communicable Diseases (obesity, CVD and type 2 diabetes). Regarding CVD, the improvement of endothelial function exerted by flavonoids, the inhibition of platelet aggregation and adhesion, and antihypertensive action could play an important preventive role. Several phytochemicals, such as flavonoids, quercetin and resveratrol, have been demonstrated to possess anti-obesity effects both in vitro and in vivo, as well as to prevent diabetes or improve glycemic control in T2D. Moreover, phytochemicals are believed to act as anti-aging agents, improving cognitive decline, and dietary flavonoids have been associated with a lower rate of dementia, mainly attributed to the antioxidant effects and to the reduction of acetylcholinesterase in the brain. In this regard, curcumin, catechin and resveratrol also showed neuroprotective effects in Alzheimer Disease. Furthermore, other protective effects of polyphenols have been described, such as those on inflammatory bowel diseases, on rheumatic diseases and on eye diseases, while the data on cancer are still conflicting [33].

## 2. Polyphenols and Obesity

Obesity is considered as a complex condition, induced by genetic, epigenetic and environmental factors and characterized by a progressive nature that negatively affects health. It is linked to excess of weight, established through a weight/square of height (body mass index, BMI) ratio ≥ 30 kg/m^2^. Obesity is linked to reduced quality of life and life expectancy [34]. Nevertheless, even people with a lower BMI but reduced lean mass and centrally deposited excess fat (visceral fat) have similar health risks, due to the alterations promoted by said type of fat [35]. Despite notions on causes and, at least in theory, methods on treatment being more advanced, the prevalence of obesity appears to be on the rise and represents one of the greatest challenges for modern medicine [36], due to its progressively heavier impact on national health systems and their economy (costs on drugs, hospitalizations and missed work shifts associated with related diseases) [37]. The existence of a weight set-point, calibrated at neuro-humoral level, makes sustained weight loss difficult over time, and causes most dietary attempts to fail [36].

The development of obesity’s phenotype determines very heavy alterations in the adipose tissue, which is no longer considered an inert store of excess calories, but as an endocrine tissue which influences the metabolism in different ways and on different systems, including the immune system [38].

These alterations, together with many other cellular mechanisms that cause a substantial increase in systemic oxidative stress mediated by reactive oxygen species (ROS), promote a chronic inflammatory state in the organism caused by redox imbalance. Therefore, systemic oxidative stress and chronic inflammation play a pivotal role in maintaining obesity and exacerbating the onset of cardiovascular complications, type II diabetes mellitus, dyslipidemia, non-alcoholic steatohepatitis and other typical conditions whose risk grows along with increasing BMI, and especially visceral fat, showed by augmented waist circumference [39].

Obesity is, at a thermodynamic level, the result of a caloric introduction greater than the number of calories expended; at a biological level, these calories can undergo a "partitioning", an addressing that favors accumulation rather than oxidation [40], which occurs more easily in an inflammatory context of insulin resistance [41,42], hyperinsulinemia [43] and oxidative stress. Dietary interventions should therefore also consider correcting oxidative stress [44].

Oxidative stress and ROS can promote food addiction, and introducing foods which lack antioxidant content could therefore facilitate hormonal alterations (reduction of leptin, increased ghrelin and alteration of their signals) which encourage weight gain or facilitate recovery from it as a result of a low-caloric diet [45].

Moreover, some evidences indicate that antioxidants could play a key role in positively modulating hormones and neuropeptides involved in appetite, satiety and regulation of energy expenditure and homeostasis (leptin and adiponectin from adipose tissue; insulin from the pancreas; GLP1, ghrelin, PYY and CCK from gastrointestinal system; NPY, AgRP and POMC/CART at the hypothalamic level), creating a less obesogenic propensity [46].

Furthermore, oxidative stress is one of the main mechanisms of obesity-related pathologies, such as atherosclerosis, type 2 diabetes, and hypertension. Oxidative stress promotes the formation of cholesterol derivatives, i.e., oxysterols, substances that are inflammatory for the endothelium of arteries and favor the formation of atherosclerotic plaques. Cell models show that phytochemicals can counteract this process [47].

The interaction between plant-derived antioxidants and possible prevention, or even reversal, of obesity and many other chronic diseases is currently being discussed [33].

On a nutritional level, a vegetable-based diet, in addition to a facilitated weight loss, thanks to its lower calorie content, caused by a greater difficulty in "extracting" calories, promotes satiety, mitigates glycemic impact, activates the ileal brake and provides prebiotics to the intestinal microbiome. Moreover, the above said diet has the potential to treat and promote the improvement of chronic diseases related to obesity, such as hypertension, atherosclerosis [48] and diabetes [49].

On the other hand, trials on human subjects encourage the use of antioxidants in obesity and demonstrate their beneficial effect, particularly for their ability to activate β-oxidation processes, induce satiety, stimulate energy expenditure, inhibit the differentiation of adipocytes, promote adipocyte apoptosis, increase lipolysis and improve lipid metabolism disorders [50].

One of the mechanisms that seems to favor the accumulation of ectopic fat—hepatic fat, in particular—through a Western diet involves altered autophagy and non-replacement of defective mitochondria. The accumulation of malfunctioning organelles can reasonably induce hepatic lipotoxic lesions and progression of steatosis, and it is notoriously linked to oxidative stress and excess ROS [51].

In fact, a mechanism that can bind polyphenols to reduced risk of obesity or favor weight-loss therapy is the stimulation of mitochondriogenesis and reduction of mitochondrial ROS. Thanks to these activities, cells produce energy with greater efficiency, and the replacement of defective mitochondria (notoriously associated with aging diseases) promotes energy expenditure, increasing lipid oxidation and promoting thermogenesis [52]. Thermogenesis could also be promoted by inhibiting noradrenaline degradation at the BAT-synapse level, due to the fact that the COMT enzyme, which catabolizes this catecholamine, is inhibited by some polyphenols [53].

Several flavonoids isolated from fruit and plant extracts have shown potent anti-obesity activity in vitro and in vivo [33]. For example, genistein and resveratrol [54] quercetin, EGCG [55], naringenin, allicin, luteolin and kaempferol showed anti-adipogenic activity [56]. Unfortunately, most of these effects have only currently been registered in cellular or animal models.

Flavonoids also stimulate autophagy, a cellular function that allows aged and altered structures to be removed, which plays an important role in lowering inflammation, which is worsened by aging and obesity [57].

In addition, experimental studies have shown that flavonoids can reduce postprandial blood glucose by inhibiting digestion and transport of glucose in the small intestine, by increasing the disposal of glucose into tissues, and by protecting and regenerating damaged beta cells and/or by improving pancreatic insulin secretion. It is likely that some specific flavonoids have a more effective action against glucose metabolism than others, and therefore it is necessary to identify their effectiveness both individually and synergistically [58].

Moreover, in a large cohort of adult subjects, a high intake of dietary flavanols has been shown to be inversely associated with blood-pressure values, an effect comparable to a good adherence to the Mediterranean diet or a moderate salt reduction: this association was stronger in hypertensive subjects compared to normotensive subjects [59].

Several other phytochemicals possess different anti-obesity properties. Chlorogenic acid and, to a lesser extent, caffeic acid significantly reduce body weight, visceral fat mass and plasma leptin, as well as insulin levels in high-fat-induced obese mice that were compared to the control group [60]. Recently, green coffee, rich in chlorogenic acid, has been associated with weight loss, greater in overweight than in normal-weight subjects [61]. The effect is probably due to the activation of UCP1 via PGC-1α [62].

Curcumin, a polyphenolic natural compound contained in *Curcuma longa,* a member of the ginger family, and consumed as a spice, may also have anti-obesity effects, both preventing preadipocyte differentiation and reducing the negative health effects of obesity [63], particularly in people with fatty liver, visceral fat and abdominal obesity. In these patients, curcumin supplementation has been shown to significantly reduce both BMI and waist circumference. Besides the known antioxidant and anti-inflammatory activity, several other mechanisms have been proposed for explaining the beneficial effects of curcumin in obesity, among which the inhibition of 11β hydroxysteroid dehydrogenase-1 expressed in adipocytes could be responsible for the reduction of visceral adipogenesis caused by the local production of cortisol, while the increased levels of the anorexigenic peptide nesfatin-1 recently described, could negatively affect food intake [64].

Although there are only a few human studies and clinical trials, also quercetin, contained in plant-based foods such as onions, garlic and ginger, as well as in apples and wine, if supplemented in overweight and obese, has been shown to reduce waist circumference and triacylglycerol levels and to lower blood-pressure values.

On the contrary, resveratrol, mainly contained in red grapes, but also in other plant-based foods, despite having been found to exert several potential anti-obesity effects in animal models, in the limited clinical trials in obese patients, showed inconsistent results. Among foods, tea and coffee, fruits, particularly berries and citrus, some spirits (red wine and beer), cocoa and nuts are the richest in polyphenols and have the highest antioxidant power. EGCG from green tea showed good effects on anthropometric measures (body weight and waist circumference). Citrus flavonoids promote an anti-inflammatory effect, while berries showed several anti-obesity effects. Apples can boost antioxidant properties via many phytochemicals like hydroxycinnamic acids, flavonols, dihydrochalcones and anthocyanidins. Onions typically contain quercetin, whose anti-obesity effects have already been described. Soybeans have widely outlined health effects, thanks to their isoflavone content (genistein, daidzein and glycetin), which modulates hormones and cytokines levels, like leptin and TNFα [50].

Taken together, these data indicate that antioxidant phytochemicals both from foods and supplements could be good candidates for the prevention and treatment of obesity through direct inhibition of adipogenesis and anti-inflammatory and antioxidant actions [33].

### AGEs

Advanced glycation end products (AGEs), products of glycation that occur in cooking at high temperatures in the presence of carbohydrates and some amino acids, are known to play an important role in the development of diabetic cardiovascular complications and altered energy metabolism, reducing energy expenditure [65]; in general, they are involved in pathologies which manifest through aging (cancer, neurodegenerative diseases, etc.), and they contribute in altering cellular functioning [66]. Through the RAGEs receptor, they activate NFkB, a cellular mediator of inflammation [67], thus stimulating and supporting the inflammatory process.

The major sources of AGEs are grilled meats, baked goods, dairy products and processed food in general: high circulating levels of them are linked to oxidative stress and inflammation, which promote aging [68]. Fruits, vegetables and whole grains appear to have less AGEs [67].

It has been observed that the increase or the prevention of AGEs’ formation has been able to modulate fatty liver disease progression in an animal model, suggesting a potential role of dietary manipulation of AGEs content by food selection and cooking methods in improving obesity and related diseases [69].

Plant foods have, together with the use of moderated cooking temperatures, a fundamental role in inhibiting AGEs production through different methods such as inhibition of ROS, dicarbonyl trapping, antioxidants activation and breaking the crosslinking of proteins [70].

Polyphenols reduce the formation of AGEs, increase their degradation, and reduce the expression of the AGEs receptor and its signal, thus contributing to slowing the disease’s development down, and, although this must be proven in intervention studies, it is already evident considering retrospective ones. Regarding the inhibition of AGEs formation, several mechanisms have been proposed: (i) blocking sugar attachment to proteins, (ii) inhibiting the formation of Schiff-base at early stage of the Maillard reaction, (iii) preventing the formation of intermediate Amadori products, (iv) reducing glycoxidation and oxidative stress by scavenging some intermediates produced during glycation reaction (e.g., free radicals, reactive dicarbonyls and nitrogen species) and (v) breaking down the synthesized AGEs crosslinks [71].

Along with antioxidants, vitamin B6 also plays a role in increasing the degradation of AGEs, and its deficiency favors the onset of diabetes. It acts as ROS scavenger, inhibits post-Amadori reactions and degradation to glyoxals, and chelates metals, thus reducing protein glycation processes and AGEs formation [72].

Marinating in vinegar or lemon also reduces AGEs [69,73]. Among spices, cloves [74], cumin [75], coriander and cinnamon [76], curcumin [77], Jamaican pepper and oregano [78] have strong inhibitory influence in AGEs production, due to the presence of phenolic compounds [79].

## 3. Polyphenols and Inflammation

Several in vitro and in vivo studies have investigated the anti-inflammatory activity of polyphenols, suggesting their ability to act on different targets, including inhibition of COXs, phospholipase A2 and lipoxygenases, which result in decreased synthesis of prostanoids and leukotrienes. Moreover, further targets of the anti-inflammatory activity of polyphenols include phosphodiesterase, kinases and transcriptases [80].

**Figure 3:**
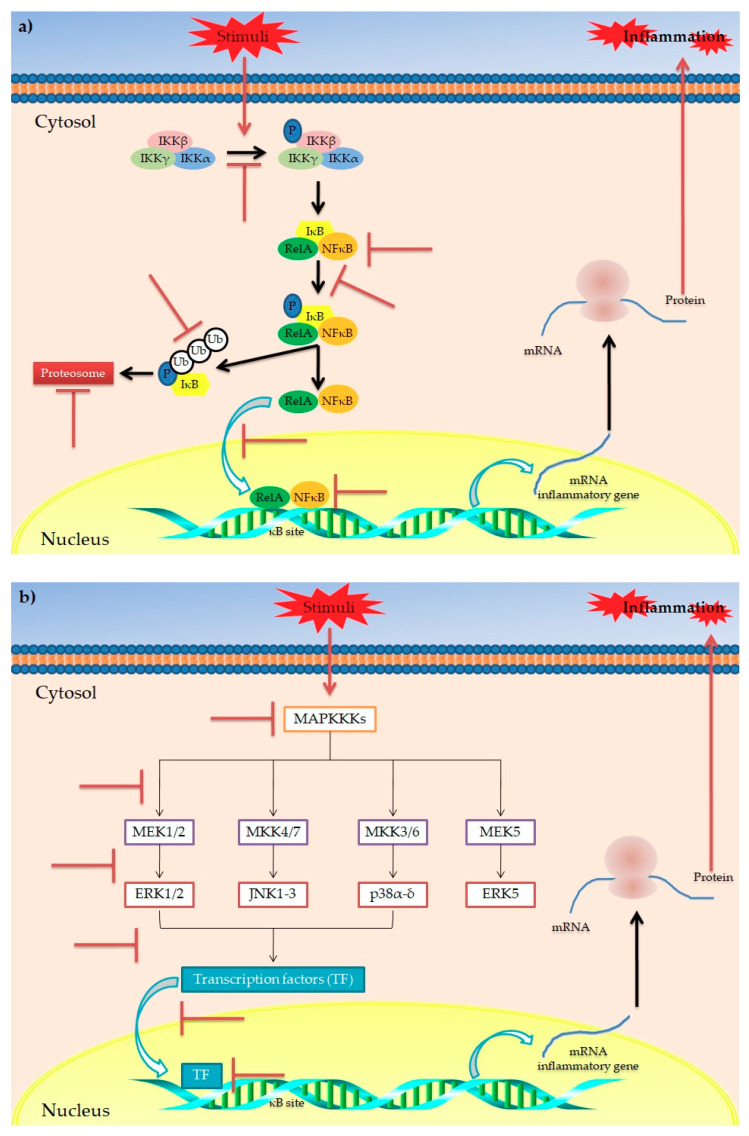
Main mechanisms of action for the anti-inflammatory activity of polyphenols in obesity. Schematic representation of the main putative targets of polyphenols in IκB/NFκB (**a**) and MAPK signaling pathways (**b**) involved in the inflammatory response. The symbol 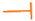 indicates the targets inhibited/blocked by polyphenols.

However, besides polyphenols’ generic anti-inflammatory potential, evidence has highlighted their role in modulating the obesity-related inflammatory status. Among the various metabolic disorders, obesity is undoubtedly the main disorder strongly related to a chronic low-grade inflammation [81,82]. This is evidenced by increased circulating levels of inflammatory biomarkers in obese patients compared to non-obese subjects, mainly released by monocytes, macrophages and dysfunctional adipocytes [83,84]. At molecular level, this is the result of an NF-κB-induced expression of pro-inflammatory genes. More specifically, NF-κB is a family of transcription factors (including NF-κB1 and NF-κB2) [85] normally present in cytoplasm and associated with IκB, a regulatory protein family that includes IκBγ, IκBβ, IκBα, IκBε and Bcl-3 [86]. When bound to IκB, NF-κB is maintained in an inactive state. In response to various stimuli (i.e., increasing oxidative stress and/or inflammation), specific genes such as NF-κB-inducing kinase (NIK), mitogen activated protein kinase kinase (MEKK), interleukin-1 receptor-associated kinase (IRAK), TNF receptor-associated factor (TRAF), PKC, and VCAM promote the activation of IKK which, in turn, phosphorylates IκB, resulting in release of NF-κB [87]. Once released, NF-κB translocates into the nucleus, inducing the expression of pro-inflammatory genes [85,88]. In this context, evidence has demonstrated the ability of polyphenols to modulate the NF-κB pathway, acting at different levels [80,89] (Figure 3a). In particular, mechanistic studies have reported that polyphenols are able to (i) counteract the activation of IKK (i.e., EGCG, epicatechin and flavonoids) [90,91,92]; (ii) inhibit the phosphorylation of IκB (i.e., EGCG, quercetin, apigenin, silymarin, kaempferol and isoliquiritigenin, curcumin) [63,80,93,94,95]; (iii) inhibit the degradation of IκB (EGCG, epicatechin, quercetin, apigenin and isoliquiritigenin) [80,90,91,92,96]; (iv) inhibit the nuclear translocation of NFκB (quercetin and isoliquiritigenin) [80,96]; and (v) block the DNA binding of NFκB (EGCG, epicatechin, quercetin and apigenin) [80,92,97,98].

Another interesting pathway involved in inflammatory response is the so-called “mitogen-activated protein kinases (MAPKs) cascade” [99,100,101] (Figure 3b). MAPKs are proteins belonging to Ser/Thr kinases, which regulate various cellular processes via modulation of gene expression in response to specific stimuli [89]. In mammals, four groups of MAPKs have been identified: extracellular signal-related kinases (ERK)-1/2, c-Jun amino-terminal kinases (JNK)-1/2/3, p38-MAPK(α-δ) and ERK5. Each one is activated via phosphorylation by specific MAP kinase kinases (MAPKKs) (MEK1/2, MKK4/7, MKK3/6 and MEK5, respectively). Each MAPKK is, in turn, phosphorylated and activated by MAP kinase kinase kinases (MAPKKKs) [102]. It has been reported that polyphenols play a role in modulation of the MAPK pathway, acting at different levels [103]. In particular, polyphenols such as kaempferol, chrysin, apigenin, luteolin, quercetin, catechin, cyanidin-3-O-glucoside, EGCG have shown to exert inhibitory activities on different MAPKs of the MAPK cascade, resulting in reduced transcription of inflammatory cytokines [80,89].

This evidence is corroborated by in vivo studies (both animal-based studies and clinical trials) which demonstrate that chronic administration of various polyphenol compounds (such as quercetin, curcumin, resveratrol, gingerols, isoflavones, procyanidins and oleuropein) significantly reduced the levels of pro-inflammatory biomarkers, including TNFα, IL-1, IL-4 and INFγ. Interestingly, the expression of these pro-inflammatory genes is mainly mediated by both IκB/NFκB and MAPK signaling pathways [80,104,105,106,107]. This suggests that the ability of polyphenols to modulate these two key pathways represents one of the main mechanisms of action for their anti-inflammatory activity. Although promising, results from in vivo studies and, in particular, clinical trials are somewhat contradictory, probably due to the study design and/or the outcomes evaluated. However, two interesting meta-analyses reported a significant anti-inflammatory effect of resveratrol [108,109]. Haghighatdoost and colleagues analyzed 15 trials involving 658 adults observing that resveratrol significantly reduced serum TNFα (young: WMD = −0.34; 95% CI: −0.57, −0.12; I2 = 60.5%; *p* = 0.038; obese: WMD = −1.52; 95% CI: −2.87, −0.16; I2 = 74.1%; *p* = 0.004) and C-reactive protein (CRP, WMD = −0.54; 95% CI: −0.78, −0.30; I2 = 77.7%; *p* < 0.0001) [108]. Similar results were reported by Koushki and co-workers, who analyzed 17 trials involving 736 subjects; the authors observed a significant TNFα- and hsCRP-reducing effect of resveratrol (TNFα: WMD, −0.44; 95% CI, −0.71 to −0.164; *p* = 0.002; Q statistic = 21.60; I2 = 49.1%; *p* = 0.02; hsCRP: WMD, −0.27; 95% CI, −0.5 to −0.02; *p* = 0.033; Q statistic = 26.95; I2 = 51.8%; *p* = 0.013) [109]. Interestingly, both the meta-analyses observed no significant effects of resveratrol in reducing IL-6 levels [108,109].

## 4. Polyphenols and Gut Microbiota

The human intestinal tract (GIT) hosts a complex microbial community, known as gut microbiota (GM), that plays an essential role in promoting its host’s health. The GM is constantly exposed to the influence of various factors, among which diets are considered one of the main key modulators, capable of strongly influencing host homeostasis through dietary metabolites derived by microbial fermentation [110]. Plant-based food models have been shown to be particularly beneficial for health. It has been well documented, regarding the Western diet, that vegetarians compared to omnivores have a lower risk of developing various chronic diseases, including obesity, hypertension, diabetes, dyslipidemia and some forms of cancer; on the other hand, unhealthy eating habits could rapidly reduce microbial diversity and promote the expansion of specific bacterial taxa, a condition known as "intestinal dysbiosis", which has been associated with gastrointestinal diseases, as well as extra-intestinal metabolic disorders, such as obesity and diabetes [111]. Understanding the interactions between food components, host and microbiota could strongly promote the possibility of targeted dietary interventions and nutritional therapies as a prevention or therapeutic strategy for microbiota-associated diseases. Recently, interactions between phytochemicals and GM, as an important contribution to the health of many plant-based foods, have been included in this context. Among these, polyphenols are interesting compounds, as they could play a role in the treatment of obesity and related metabolic diseases [112]. It has been reported that some polyphenols possess important biological properties, including antioxidant, anticarcinogenic or antimicrobial activities against pathogenic microorganisms [113,114,115]. Monagas et al. have also reported potent anti-inflammatory activities in vitro of bioactive metabolites of proanthocyanidins, produced by microbial metabolism; specifically, the downregulation of cytokines TNF-α, IL-1 and IL-6 induced by lipopolysaccharides, in peripheral blood mononuclear cells [116]. Recent evidence has associated long-term dietary intake of polyphenols with greater protection against chronic degenerative diseases such as cancer, neurodegenerative and cardiovascular diseases [117], as well as the improvement of insulin resistance, glucose tolerance and anti-obesity [118]. However, it is important to consider that the responses to consumption of dietary polyphenols are closely associated with interindividual variability [119]. The bioactivity of polyphenols is mediated by a series of metabolites produced by the colonic microbiota, since most of them are ingested as non-absorbable precursors, whose bioavailability is related to both chemical structure and rate of absorption [120]. Food polyphenols, in fact, have a very low bioavailability. Only a small percentage of dietary polyphenols is absorbed in the small intestine [121]; most of them reach the colon, where they are broken down by intestinal bacteria to produce a series of metabolites [122]. Simple polyphenols, such as aglycones, are directly absorbed by the intestinal mucosa at small intestine level; on the other hand, glycosides, which are structurally more complex, are only partially absorbed at small intestine level after microbial deconjugation, while most of them reach the large intestine, similarly to what happens to polyphenolic polymeric forms. The latter, being the most structurally complex, are rarely absorbed by enterocytes in the small intestine and reach the colon. Here, they can be absorbed through the epithelium, diffused into the blood stream or metabolized by intestinal microbes through hydrolysis, cleavage and reduction reactions, resulting in the production of bioavailable metabolites [123,124,125]. Interestingly, bioactive forms of some polyphenolic metabolites, being more effective and better absorbed than the corresponding native precursor, have been documented, which further demonstrate the essential contribution of microbial catabolism in modulating the effects these polyphenolic compounds have on health [126]. However, the ability of individuals to obtain more bioactive polyphenolic derivatives, or, in other words, the final health effects of dietary polyphenols, is related to the richness and biodiversity of intestinal bacterial taxa. An example is provided by daidzein, an isoflavone subject to two different metabolic pathways in relation to intestinal microbial phenotype most representative of the subject. The activity of *Streptococcus intermedius*, *Bacteroides ovatus*, *Bifidobacterium* spp., *Ruminococus productus*, *Lactobacillus mucosae*, *Lactococcus garvieae*, *Enterococcus faecium*, *Veillonella* spp., *Eggerthella* spp. YY7918, *Slackia* sp. HE8, *Finegoldiamagna*, *Slackia equolifaciens* (Strain DZE) led to the production of (S) -equol in some subjects; while *Clostridium* spp. produced O-demethylangolensin (O-DMA) in others [127]. (S) -equol exhibited high antioxidant activity and prevented cellular oxidative damage; while O-desmethylangolensin has shown to be associated with obesity [128].

Although further studies are needed, some of the microorganisms and the enzymatic systems involved in the microbial catabolism of polyphenolic compounds have been identified [129]; however, it is necessary to clarify the impact of polyphenols in modulating the composition of human GM, and the possible antimicrobial and prebiotic effects. As for the latter, it should be noted that most studies on the prebiotic effect of dietary polyphenols have been conducted by testing foods rich in polyphenols rather than specific classes of these compounds. An increase in *Lactobacillus* spp. and/or *Bifidobacterium* spp. has been consistently observed for several types of food containing different classes of polyphenols; specifically, in response to fermentation of pure fruit or fruit rich in anthocyanidins both in vitro and in animal models [130,131], after the consumption of tea polyphenols [132], coffee [133,134], strawberries, raspberries and blackcurrants [135], cocoa and chocolate in animal models [136,137] and in human intervention studies [138,139] and flavonoid-enriched apples in mouse studies [131]. Furthermore, in mice fed a high-fat diet, freeze-dried mango prevented the reduction of *Bifidobacterium* [140].

On the other hand, studies on specific bioactive metabolites showed that the anthocyanins and their tested derivatives positively and significantly influenced the growth of beneficial bacterial genera such as *Lactobacillus* spp., *Enterococcus* spp. and *Bifidobacterium* spp. [141]; resveratrol stimulated the growth of *Bifidobacterium* and *Lactobacillus* of the GM of mice instead [142,143]. The growth of *Bifidobacterium* and *Lactobacillus* was also stimulated in vitro by pomegranate ellagitannins [144]. Studies also conducted in rats indicated that quercetin intake alleviated intestinal microbial dysbiosis induced by the consumption of a high-fat diet [145].

Concerning the antimicrobial activity of polyphenols, different ways of action have been described. Quercetin, which belong to the flavonoid family, showed protective activity against *Shigella* infection in animal models [146], the ability to inhibit *Escherichia coli* gyrase by acting on the ATPase GyrB subunit [147] and antiviral activity by enhancing the action of acyclovir against *Herpes simplex virus* (HSV) infection [148]. In vitro assays have shown that quercetin induced an increase in the permeability of bacterial membrane to ions and, consequently, the dissipation of the membrane’s potential and loss of cell motility [149]. Some polyphenolic metabolites, such as catechin epigallocatechin-3-gallate (EGCG), exhibited antibacterial activity by inhibiting biofilm formation, or by binding and ionizing staphylococcal enterotoxin B [150,151]. EGCG was also capable of damaging the lipid layer of the bacterial cell membrane and its toxic action against various pathogenic microorganisms such as *Streptococcus pyogenes*, *Bacillus* spp. and *Clostridium* spp., *Salmonella typhi* and enterohemorrhagic *E. coli* has been demonstrated [152]. It was shown that polyphenolic bioactive compounds could have an indirect antimicrobial effect by acting directly on the immune system and leading to an increase in cytokine response [153]. Multiple mechanisms in which EGCG had specific antiviral effects have also been highlighted, e.g., preventing virus absorption to cells, viral replication (by inhibiting viral polymerases) or inhibiting the fusion mechanism of cell and viral membranes [152].

Despite compelling evidence of beneficial health effects related to the consumption of plant-based foods, future research should aim at improving the understanding of interactions between dietary phytochemicals and gut microbiota, in order to identify novel polyphenol-derived microbial bioactive metabolites, and, above all, to define prebiotic effects on the GM for their potential therapeutic/nutraceutical use. The latter is hampered by the low bioavailability of polyphenols and their inability to reach target sites efficiently; for this reason, various approaches have been developed in recent years, aimed at improving the effectiveness of polyphenols release locally in the GIT and reducing their systemic diffusion. Among the suggested strategies we found prodrugs, osmotic controlled or pH-sensitive release systems and enzymatic linkers [117], which have paved the way for an efficient use of phenolic compounds also as a possible preventive purpose towards disorders characterized by a dysbiotic phenotype.

## 5. Conclusions and Future Perspectives

This review aims at elucidating the reciprocal interactions between GM, inflammation and obesity, and the potential contribution of a plant-based diet in these conditions. Plant-based diets can improve inflammation, obesity, and microbiota because of their higher contents of polyphenols. Modern nutritional national guidelines underline the importance of vegetable foods, particularly in their unprocessed status [154,155].

Recently, plant-based diets have been linked to a lower expression of inflammation markers [156], reduced risk of diabetes [157], hypertension [158], cancer and cardiovascular diseases [159] and are recommended for steatohepatitis management [160].

It is very likely that polyphenols in vegetables contribute to these effects, due to their anti-inflammatory properties, their effect on microbiota and the physiology of their body composition.

**Figure 4:**
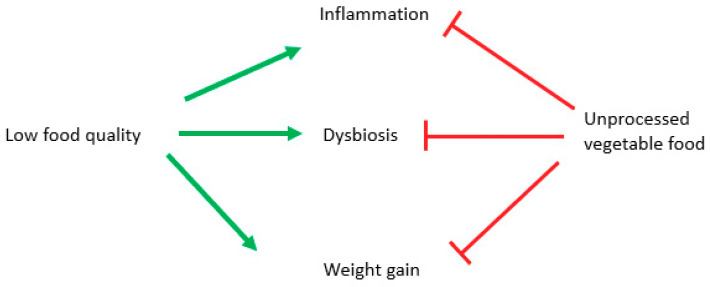
Potential role of diverse diet styles on inflammation, obesity and microbiota. Green arrows stimulate, and red lines block.

Although many cellular mechanisms have been proven to be effective only in animal and cellular models, anti-inflammatory, prebiotic, and anti-obesity effects of plant-based diets have been demonstrated by observational and intervention studies, which showed connections between better health and a longer lifespan and a consistent use of unprocessed vegetables foods. The effect is due to fibers [161], mineral salts like potassium and magnesium [162] and antioxidants [163] (Figure 4).

Antioxidants play a fundamental role in health, and their introduction is necessary to prevent and treat or manage non-communicable diseases related to low-grade basal inflammation and dysbiosis, typical of conditions such as overweight or obesity. The best way to ensure a wide and varied introduction of antioxidants is a plant-based diet, which does not necessarily imply eliminating foods of animal sources; instead, it guarantees that vegetables be preponderant among other foods, as it occurs in most diets which are considered healthier (Mediterranean, DASH and similar). While data from RCT are still limited, guidelines around the world, including recently published American ones [164], recommend that antioxidant-rich plant foods should prevail, going far beyond the equalization of calories from unprocessed and processed foods.

Inflammation, a preserved mechanism of defense from various threats, like pathogens, trauma, toxic substances, etc., is the main cause of conditions linked to aging, such as cardiovascular diseases, cancer, etc. [165]; moreover, obesity also induces a low-grade inflammation.

Thus, the hypothesis is that polyphenols can prevent, attenuate and even reverse inflammation and its related (dependent or caused by) conditions like obesity and dysbiosis. Good bacteria are fed by antioxidants and fibers [117] and they have their own antioxidant properties, such as chelating metals, modulating their host’s antioxidant defense, and producing vitamins and their enzymes like catalase and SOD [166].

Furthermore, microbiota contributes to the detoxification processes through vitamin and enzyme production [167], and toxicants can be a source of inflammation and dysmetabolism [168]. Recent evidence reported peculiar characteristics of GM and the impact of the Mediterranean diet in Italian obese people [169,170].

Unfortunately, a major concern is understanding which condition comes first, obesity, dysbiosis or inflammation, as these factors reciprocally interact to prepare the field for pathological chronic conditions.

We can identify/classify various situations in which we see the following:

(1) Dysbiosis is present, although often transiently, from birth, because of caesarean section, missing breastfeeding, altered maternal microbiota and antibiotics abuse;
(2) Dysbiosis comes after shifting to a low-quality diet;
(3) Obesity is caused by overfeeding in quality and favored by low food quality;
(4) Obesity, especially visceral one, causes inflammation due to its hormonal alterations (leptin, TNFα, low adiponectin, etc.);
(5) Inflammation is driven by diet composition.

These factors interact to support disease(s).

The relationship between inflammation and a low-quality diet is well-known, as well as a good intake of vegetables, is associated with an anti-inflammatory effect [171].

Anti-inflammatory and antioxidant effects of plant-based diets have been established by recent meta-analysis [172], while low-quality diets, represented by a high glycemic index, or excessively refined diets, are linked to higher mortality [173], lower levels of micronutrients and a higher risk of obesity [174] (Figure 4).

A high-quality diet can counteract metabolic endotoxemia, represented by the presence of LPS and other bacterial metabolites which stimulate inflammatory response and might be a plausible cause of metabolic and cardiovascular diseases [175].

Furthermore, polyphenols reduce cardiovascular risk by improving the reverse transport of cholesterol, by increasing the removal of cholesterol from macrophages in atherosclerotic plaques (through LXR) and increasing the efficiency of HDL (good cholesterol), which brings cholesterol back to the liver. They also protect lipoproteins from oxidation [176].

Can everyone take advantage of an increase of vegetables?

The effect is subjective because of the differences in basal conditions of dysbiosis, persistency of variation in microbiota induced by diet, etc.; consequently, the outcome of the diet can be extremely different for each individual [177].

Regarding GM composition, *P. copri* can be responsible for the cardiovascular outcome of a Mediterranean diet, and, in particular, its abundance reduces CV protection [178].

Moreover, baseline microbiota can influence weight-loss maintenance [179], and the metabolism and absorption of polyphenols are influenced by microbiota and genetics [180].

Therefore, it is clear that only a sustained diet rich in antioxidants can modulate inflammation, microbiota and oxidative stress in order to imprint a consistent and durable effect on the body and its metabolism [179].

On the other hand, Western and poor diets have showed an “antibiotic effect” on GM, thanks to lack of fibers, food additives, plastics residues, etc. [181,182], and for this reason, we are observing a disappearance of good microbial species [183], which, in turn, causes modern diseases.

AGEs are another feature of Western-type diets. Their reduction by lowering cooking temperature, the use of some spices and abundance of vegetables should be a part of a dietary treatment [184].

Vegetable foods can improve inflammation, obesity and microbiota in various ways.

Microbiota are driven primarily by diet, and vegs favorite good bacteria, which influence antioxidant capacity, degrade toxic metabolites and allow for the synthesis of SCFA, which are very important metabolites in the control of blood pressure, atherosclerosis, hunger, metabolism and immunity [185].

Inflammation is modulated by several components of vegs, which contain various substances with antioxidant properties. They reduce activation of inflammatory pathways, dependents from oxidative stress. Inflammation is one of the causes of insulin resistance and fat accrual [41]; thus, we can speculate that reducing inflammation could reduce the propensity for fat mass expansion and ease lipolysis and oxidation of fats.

Obesity can be counteracted by many chemicals presents in vegs. It is expected that these components act synergistically to favor energy expenditure and to reduce orexigenic signals, supporting a negative energy balance without stimulating an excessive famine response (main factor of weight regain in normal hypocaloric regimens), which allows the individual to maintain a lower weight. [186].
